# Investigation on the Influencing Factors Related to Quality of Life of Adult Epilepsy Patients in Wenzhou, China, Based on Structural Equation Model

**DOI:** 10.1155/2022/4336622

**Published:** 2022-09-28

**Authors:** Huijing Sun, Ling Chen, Kelong Chen, Bin Li, Zhimin Wu

**Affiliations:** Department of Neurology, Wenzhou Hospital of Traditional Chinese Medicine, Wenzhou 325000, China

## Abstract

**Objective:**

The aim of the study is to investigate the influencing factors of quality of life in adult patients with epilepsy in Wenzhou in China.

**Methods:**

A total of 190 patients who visited our hospital from July 2019 to February 2021 were included in the study. Demographic data and disease status were collected. Moreover, QOLIE-31, PSQI, ESS, HAMD-17, and GAD-7 were used in the questionnaire survey. Structural equation model fitting was used to analyze the influencing factors of quality of life in adult patients with epilepsy.

**Results:**

The scores of the dimension of onset worry in men were greater than those of women (*P* = 0.049), and the scores of the dimension of life satisfaction were lower than women (*P* = 0.047). The scores of cognitive function decreased with age (*P* = 0.047). The scores of quality of life of unemployed and drinking patients significantly decreased (*P* < 0.05). The score of quality of life positively correlated with good economic status and family relations (*P* < 0.05). The score of emotional health increased first and then decreased with the course of the disease. With the decrease in seizure frequency and the extension of months without a seizure, the score of quality of life gradually increased. Furthermore, the structural equation model showed that health status was directly correlated to the quality of life of patients with epilepsy.

**Conclusion:**

Male, unemployment, drinking, older age, and disease are negatively related to the quality of life in patients with epilepsy. However, good economic status, good family relations, and good colleague relationships are positively related to the quality of life.

## 1. Introduction

Epilepsy is a kind of disease with short-term abnormal brain function, which has a high incidence rate. The clinical manifestations include major attack, minor attack, psychomotor, limited, and mixed attack [1]. Human beings have experienced a long history to discover and understand epilepsy. The pathogenesis of the disease is complex, and the clinical manifestations are diverse. According to different pathogenesis, the concept of clinical treatment changes. More than 70 million patients with epilepsy in the world are mainly concentrated in economically poor areas [2]. The total number of epileptic cases in China is about 9 million, and there are about 400,000 new epileptic cases every year [3]. Because the disease is a chronic disease that can last for decades, it has a great impact on the patient's physical, mental, marital and economic, and social status.

Compared with the general population, patients with epilepsy have a significantly higher rate of depression. A meta-analysis showed that the incidence of depression in patients with epilepsy was 23.1%, more than twice as high as the general population [4]. More importantly, anxiety and depression increase the risk of suicide.

There is a correlation between the sleep cycle and the onset time and frequency of epilepsy. Lack of sleep can affect seizures, and seizures can also affect sleep structure. A clinical trial study found that 21% of patients with epilepsy had seizures at night, 42% had seizures during the day, and 37% had seizures during the day and night [5]. Therefore, in the clinical treatment of epilepsy, paying attention to sleep is of great significance for controlling epileptic seizures and improving the quality of life of patients.

In clinical work, whether epilepsy is cured and rehabilitated is based on whether epilepsy is controlled or not. Although a variety of new antiepileptic drugs that can control most seizures have been put on the market, there are still some epilepsy that cannot be controlled by drugs. The long-term use of antiepileptic drugs and their side effects not only bring serious psychological burdens to the patients themselves but also cause psychological pressure and heavy economic burden to the families of patients with epilepsy. In 1993, WHO defined quality of life as “the experience of individuals in different cultures and value systems based on life expectation and state, including physical, psychological and whole social adaptability” [6]. Therefore, the clinical treatment of epilepsy is no longer limited to etiological treatment and symptom relief but to making the patients recover to a healthy level.

In recent years, there are more researches on the influencing factors of the quality of life in patients with epilepsy. At present, the investigation and research on the influencing factors of the quality of life of adult epilepsy patients in Wenzhou has not been reported. Therefore, the purpose of this study was to explore the influencing factors of quality of life in adult patients with epilepsy in Wenzhou in China.

## 2. Subjects and Methods

### 2.1. Subjects

A total of 190 patients who visited our hospital from July 2019 to February 2021 were included in the study. All patients were diagnosed and treated by neurologists. The inclusion criteria are as follows: (1) patients who met the epilepsy diagnostic criteria of the “Seizure Classification and Diagnosis Essentials published by the International Anti-Epilepsy Alliance in 2010,” (2) the cause and type of seizures in patients with epilepsy that were not limited, (3) the patients whose age range was 18 to 75 years old, without restriction on men and women, (4) the patient who had an education level of elementary school or above and can read and complete the self-rating scale, and (5) the patients who volunteered to participate and sign informed consent. The exclusion criteria are as follows: (1) patients with a history of other psychotic symptoms such as delirium; (2) patients with progressive central nervous system diseases such as acute stroke, brain malignant tumors or metastases, various encephalitis, and acute phases; (3) patients with Parkinson's disease and the like; (4) patients with a history of malignant tumors; (5) patients with a history of serious systemic diseases such as severe heart, lung, liver, kidney, and blood system; (5) patients with cognitive dysfunction, and (6) patients who refused to participate in the study.

### 2.2. Questionnaire Investigation

The patients with epilepsy who met the inclusion criteria were investigated by questionnaire. First of all, all the patients who participated in the study were informed of the purpose of the study and the principle of confidentiality of the psychological evaluation results. The patients were assessed and investigated voluntarily. The researcher will explain the content of the scales and answer the questions. The general information and forms were filled out by the patients themselves. After the completion of the questionnaire, the neurologist scored each scale according to the standard score of each scale.

### 2.3. General Clinical Data Collection

The demographic and clinical data of patients with epilepsy were collected through self-made general survey forms. Demographic data included age, gender, marital status, education level, smoking and drinking history, family economic status, family relationship, colleague relationship, occupation, and so on. The clinical data included the time of the first diagnosis, age of first seizure, course of the disease, seizure frequency, types of drugs taken, main types of epilepsy, intractable or not, months without seizure after treatment, and so on.

### 2.4. QOLIE-31

The American quality of life in epilepsy (QOLIE-31) was used to evaluate the quality of life, which was compiled by Cramer and other experts in 1998 [7]. QOLIE-31 consisted of seven factors, such as seizure anxiety, life satisfaction, emotional health, energy fatigue, cognitive function, drug effect, and social activities. The total item was the overall health level, including 31 items. The score of the scale was calculated by “doctor Tong” in the mobile app. According to the scoring rules of the scale, the item of overall health level was not included in each factor score or total score, so it was listed separately for statistics. In this study, the score of each factor was the original score converted into the percentage score, which was not multiplied by the weight.

### 2.5. HAMD-17

The Hamilton depression scale-17 (HAMD-17) was used to evaluate the depression level, which was compiled by Hamilton in 1960 [8]. HAMD-17 consisted of 17 items. Most of the items were scored at 5 levels of 0–4, and a few items were scored at 3 levels of 0–2. The sum of the score of each item is the total score. A score of less than 7 is normal; 7–17 may be depressed; 17–24 is depression; and more than 24 may be severe depression.

### 2.6. GAD-7

The generalized anxiety disorder-7 (GAD-7) was used to evaluate the anxiety level [9]. There were 7 items in GAD-7, and the items were scored at four levels of 0–3. The total score of GAD-7 was 21 points. The higher the score, the more serious the anxiety symptom.

### 2.7. PSQI

The Pittsburgh sleep quality index (PSQI) was used to evaluate sleep quality, which was compiled by Buysse et al. [10]. The total score of PSQI was 21 points. The higher the score, the worse the sleep quality. The scale also had seven-factor scores, including sleep quality, sleep time, sleep time, sleep efficiency, sleep disorders, sleep drugs, and daytime dysfunction. The various symptoms of insomnia patients were covered. The cutoff value was 7 points.

### 2.8. ESS

The Epworth sleeping scale (ESS) was used to assess excessive daytime sleepiness, which was compiled by Johns [11]. The total score of PSQI was 24 points. More than 6 points indicated drowsiness; more than 11 points indicated excessive drowsiness; and more than 16 points indicated dangerous drowsiness.

### 2.9. Experimental Quality Control

The case observation table designed by the designer was used to express the content of this study. Researchers truthfully recorded all data and ensured the authenticity and validity of all data. All researchers strictly abided by the research plan during the research, and uniform guidelines and evaluation standards were used. The recording and evaluation of all information were carried out in accordance with the requirements of the research protocol.

### 2.10. Statistical Analysis

SPSS22.0 and AMSO22.0 were used for statistical analysis. Quantitative data were expressed as mean ± standard deviation (SD). Independent sample *t*-test was used to compare the quantitative data between the two groups; one-way ANOVA was used to perform a comparison among multiple groups, and snk-q test was used to perform a comparison between the two groups. Qualitative data were expressed as the number or constituent ratio. Pearson correlation analysis was used to analyze the correlation between the two quantitative variables. The influencing factors of quality of life were analyzed by multiple linear stepwise regression analysis and structural equation model fitting. The difference was statistically significant when *P* < 0.05.

## 3. Results

### 3.1. The General Clinical Characteristics

A total of 200 epileptic patients participated in this study. Due to incomplete data, three patients were excluded. Four patients were excluded because the questionnaire was not submitted in time. As three patients gave up halfway, they were also excluded. The general clinical characteristics of the 190 patients are shown in Table 1. Moreover, the prevalence of epilepsy patients is shown in Table 2. Furthermore, the anxiety, depression, and sleep conditions are shown in Table 3. There were 34.7% of patients with different degrees of anxiety, 28.9% of patients with different degrees of depression, 28.4% of patients with different degrees of lethargy, and 22.1% of patients with poor quality of sleep.

### 3.2. Correlation between Quality of Life and Demographic Factors in Patients with Epilepsy

The dimensions and scale scores of quality of life in patients with epilepsy are shown in Table 4. The correlation between the quality of life and demographic factors in patients with epilepsy are shown in Table 5. The score of the anxiety dimension in males was higher than that in females (*P* = 0.049). However, the score of the dimension of life satisfaction in males was less than females (*P* = 0.047). The scores of cognitive function were decreased with age (*P* < 0.05). However, there was no significant correlation between the quality of life and the status of marriage, education level, or smoking (*P* > 0.05). Moreover, the correlation between the quality of life and occupation was mainly reflected in the dimension of seizure anxiety (*P* = 0.010). The scores of students and unemployed patients were relatively low, while the scores of other occupations were relatively high. Similarly, the scores of anxiety (*P* = 0.002) and emotional health (*P* = 0.044) of patients with alcohol drinking were higher than those of patients without alcohol drinking. The score of emotional health was positively correlated with good economic status (*P* < 0.05). There was no significant correlation between the quality of life and family relationships in the dimension of life satisfaction (*P* > 0.05). However, a good family relationship is positively correlated with the quality of life (*P* < 0.05). The quality of life of epileptic patients was significantly correlated with their colleagues and working conditions in the four dimensions of seizure anxiety, energy fatigue, social activity, and total scale (*P* < 0.05).

### 3.3. Correlation between Quality of Life and Epileptic Status in Patients with Epilepsy

As shown in Table 6, there was no significant correlation between quality of life with etiology and age of onset (*P* < 0.05). The score of emotional health increased first and then decreased with the course of the disease. The scores of cognitive function, social activity, and total scale reached the highest when the course of the disease was 6–10 years and then gradually decreased with the extension of the course (*P* < 0.05). For the type of seizure, patients with simple and complex seizures scored lower in the dimension of drug influence than those with general seizure and absence seizure (*P* < 0.05). Furthermore, with the decrease in seizure frequency, the score of quality of life increased gradually (*P* < 0.05). Moreover, with the prolongation of seizure-free months and the decrease of medication amount, the score of quality of life increased gradually (*P* < 0.05). Interestingly, the score of quality of life in patients with refractory epilepsy was lower than that in patients with nonrefractory epilepsy (*P* < 0.05).

### 3.4. Correlation Analysis of Anxiety, Depression, Sleep, and Quality of Life in Patients with Epilepsy

As shown in Table 7, Pearson correlation analysis showed that the quality of life was negatively correlated with anxiety, drowsiness, depression, and low sleep quality (*P* < 0.01). However, the quality of life was positively correlated with health status (*P* < 0.01).

### 3.5. Structural Equation Model Analysis of Influencing Factors of Quality of life in Patients with Epilepsy

**Figure 1:**
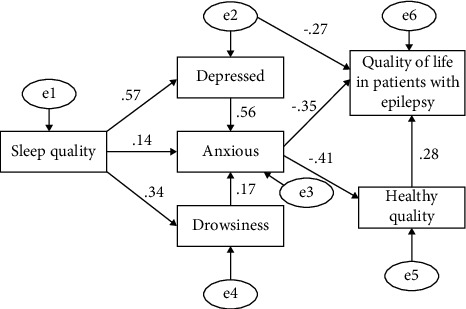
Structural equation modeling of factors influencing the quality of life in patients with epilepsy.

According to theoretical assumptions, domestic, and foreign literature and the results of Pearson's correlation analysis, after model fitting, the path that was not statistically significant was excluded, and the structural equation model established is shown in Figure 1. The maximum likelihood method is used to test each path coefficient.  Table 8 shows that the nine paths involved in Figure 1 were all statistically significant (*P* < 0.001).

In order to examine the fitting degree between the measurement data and the conceptual model and the relationship between the items, multiple indicators were used for comprehensive evaluation. The absolute fitness was evaluated by *X*^2^/DF, GFI, SRMR and RMSEA. AGFI, CFI, and IFI were used to evaluate the relative fitness. PNFI and PGFI were used as simple and effective adaptation indexes, and their values should be greater than 0.5. As shown in Table 9, most of the fitting indexes of the model were in the required range or close to the reference standard.

The model showed that health status had a direct effect on the quality of life. Anxiety and depression not only had a direct effect on the quality of life but also played an indirect role through health status and other factors. Sleep quality played an indirect role in the quality of life through anxiety, depression, sleepiness, and health status. Somnolence played an indirect role in the quality of life of epilepsy patients through anxiety and health status.

## 4. Discussion

Regarding the effect of gender on the quality of life of adult patients with epilepsy, different studies have different conclusions. Our results show that the score of men is higher than women in the onset of worry, while the score of women is higher than men in life satisfaction. Raty et al. also have found that the quality of life of females is better than that of males [12]. Another study that investigates epilepsy patients in two different places in West Africa showed that men in one place have worse quality of life than women, and in the other place, gender is not a factor influencing the quality of life of epilepsy patients [13]. The reason for the different results may be related to the differences in humanities, economy, and culture in different regions. Furthermore, our results show that the influence of age on the quality of life of patients with epilepsy is mainly manifested in cognitive function and social function. A European study showed that the quality of life of epilepsy patients gradually deteriorated with age [14]. A study of 207 epileptic patients in China also showed that age is negatively correlated with the quality of life of epileptic patients [15]. The reason may be that with the increase of age, the physical strength of the elderly is gradually weakened, or they are troubled by other chronic or acute diseases.

Our results show that marriage is not a factor influencing the quality of life of adult epilepsy patients in Wenzhou. Wang et al. found that the quality of life of married patients with epilepsy in the young and middle-aged population is better than that of unmarried adult patients with epilepsy [16]. Compared with married adults, unmarried adults with epilepsy are more anxious and depressed. In a cross-sectional study in Egypt, a total of 920 students were surveyed. Many of them have a negative attitude towards major life milestones in epilepsy, such as marriage and childbirth [17]. We do not find a difference between married and unmarried, which may be related to the small sample size of this study. It may also be that the economic situation in Wenzhou is better, and patients can use better drugs to control epilepsy, so it does not affect the patient's marriage.

Our results show that education level is not an influencing factor in the quality of life of adult patients with epilepsy. Some foreign scholars believe that education is positively correlated with the quality of life [14]. A study in China shows that higher education is a protective factor for the quality of life of epilepsy [18]. Wenzhou is the first place of the private economy, and most people are self-employed. Most can still work in their own factories even if they have a low education level. They have a good source of income and considerable economy, so they have no great impact on the quality of life. Moreover, our study shows that the association between quality of life and occupation of epilepsy patients is mainly reflected in the dimension of seizure worry, and the scores in students and unemployed patients are relatively low. It has been reported that the occupation of epilepsy patients has an important impact on their lifestyle and quality of life [19]. Because of suffering from epilepsy, patients worry about seizures without warning, so they feel no sense of security, anxiety, and fear. These reasons may lead to their deliberately reducing contact with society, resulting in their low employment rate and low quality of life.

Our study shows that smoking is not an influencing factor in the quality of life in patients with epilepsy. Dwoetzky et al. found that compared with never smoking, the incidence of convulsions is increased [20]. This is not consistent with the results of this study, which may be related to smoking cessation after the diagnosis of epilepsy. However, the sample size of the study of Dwoetzky et al. is 110, which is small, so further research is needed. Smoking is a risk factor for many diseases. Therefore, patients with epilepsy still are needed to advise to quit smoking.

This study shows that the emotional health score of patients with epilepsy is positively correlated with good economic status. Cheng Jianhua et al. suggest that the better the economic situation, the better the quality of life of patients [21]. Zhang Yuting and others also show that the economic situation and the quality of life scores of patients with epilepsy are positively correlated to life satisfaction, emotional health, and total score [15]. Our study is basically consistent with the above research results. The quality of life of patients with superior economic situations is relatively high, and they will not worry too much about living and medical expenses due to economic problems. All in all, improving the economic situation of epilepsy patients will help improve the quality of life of epilepsy patients. Moreover, we also show that good family relations are positively correlated with the quality of life. The patient spends most of his time in the family environment and has the closest relationship with family members. Therefore, the support and understanding of family members have an important impact on the physical and mental health of patients. Especially, when the patient has a seizure, it also has a significant impact on the patient's rescue [22]. Therefore, good family relationships can improve the quality of life of patients with epilepsy.

Our results show that the etiology and age of onset are not the influencing factors of the quality of life of patients with epilepsy, which may be related to the insufficient sample size or the lack of understanding of epilepsy. In the study of Yuting et al., the quality of life of patients without definite etiology is better [15]. In addition, another study shows that the younger the age of onset, the more serious the impact on the brain development process, and the more obvious the cognitive impairment, which results in the low quality of life of patients [23]. The different results of this study may be due to the fact that the sample size is mainly adults and the sample size is relatively small.

This study shows that emotional health first increases and then decreases with the course of the disease. Djibuti et al. believe that the long course of epilepsy is positively correlated with the total score of quality of life, seizure anxiety, and social activities [14]. The reason why this study is inconsistent with it may be caused by the different understanding of the disease and the different control of the attack. Moreover, we have found that patients with simple and complex seizures score lower in the dimension of drug influence than patients with generalized seizures and absence seizures. However, the investigation of Li Keng et al. on the quality of life of epilepsy patients in rural areas of Gansu Province showed that seizure type is not the influencing factor of their quality of life [24].

The results show that with the decrease in seizure frequency, the score of quality of life increases gradually. A study by Fawale shows that there is no significant difference in the quality of life between epileptic patients and normal people [25], which is consistent with this study. It can be seen that the control of seizure frequency can significantly improve the quality of life of patients with epilepsy. In addition, we have found that the score of quality of life gradually increases with the prolongation of seizure-free months after treatment. The results are consistent with the control of seizure frequency. Another study also shows that patients with poor seizure control are younger and more depressed and have a lower quality of life [26]. The results are basically consistent with this study. The results of this study show that the score of quality of life increases with the decrease in the number of drugs. A foreign study shows that multidrug combination therapy for epilepsy is helpful for disease control, but it has an impact on the quality of life [27]. Furthermore, another study also shows that the total score of quality of life and various factor scores significantly decrease when treated with multidrug combination [28].

The results show that the quality of life of patients with epilepsy is correlated with anxiety, depression, and quality of sleep. A study conducted by Wang Yingying et al. shows that patients with epilepsy have obvious somatization symptoms, accompanied by anxiety, depression, and other negative emotions, and depression and poor quality will increase the risk of suicide [29]. A study by Johnson et al. shows that anxiety and depression are independent influencing factors of quality of life in patients with epilepsy [30]. Another study also shows that anxiety, depression, and sleep disorders have a greater impact on the quality of life of patients with epilepsy than the control of short-term seizures [31]. Epilepsy patients are prone to sleep disorders, which affect their quality of life [32]. However, another study on juvenile myoclonic epilepsy shows that despite the appropriate drug treatment, the decrease in sleep quality and daytime sleepiness in patients increase the risk of seizures [33].

Nevertheless, we also explore the relationship between the quality of life of patients with epilepsy and sleep, anxiety, depression, lethargy, and health status through structural equation models. There is currently no relevant research method to study the quality of life of patients with epilepsy. This method can not only understand the direct impact of sleep, anxiety, depression, sleepiness, and health status on the quality of life of patients with epilepsy but also intuitively understand whether there is an indirect relationship between anxiety, depression, sleep, sleepiness, and health status through structural equation models.

There are also some limitations in our study. First, because this study is a cross-sectional study, we do not further explore the impact of the respective proportions of various factors on the quality of life of patients with epilepsy. Secondly, the sample size of this study is relatively small, and it is mainly for adult patients with epilepsy. It is impossible to analyze more categories for further research. In the future, more large-scale cohort studies and randomized controlled studies need to be conducted on different factors affecting the quality of life of patients with epilepsy through multidisciplinary cooperation.

## 5. Conclusion

In conclusion, the quality of life of adult patients with epilepsy in Wenzhou is affected by gender, age, occupation, economy, family relationship, work condition, course of the disease, seizure type, seizure frequency, months without seizure after treatment, whether it is refractory, and the amount of medication. Sleep disorders, drowsiness, anxiety, depression, and health status all also affect the quality of life of patients with epilepsy.

## Figures and Tables

**Table 1:** The general clinical characteristics.

Items		Cases (*n*)	Percentage (%)
Gender	Male	87	45.8
	Female	103	54.2

Age (years)	≤25	57	30.0
	26–40	91	47.9
	≥41	42	22.1

Marriage	Unmarried	64	33.7
	Married	116	61.1
	Divorce	10	5.3

Education	Below junior high school	29	15.3
	Junior middle school	63	33.2
	High school	39	20.5
	Junior college	51	26.8
	University or above	8	4.2

Occupation	Student	17	8.9
	Part-time job	22	11.6
	Full-time job	99	52.1
	Retired	6	3.2
	Unemployed	46	24.2

Smoke	No	177	93.2
	Yes	13	6.8

Drink	No	175	92.1
	Yes	15	7.9

Economic situation	Good	23	12.1
	Secondary	111	58.4
	Difference	56	29.5

Family relations	Harmonious	113	59.5
	Commonly	64	33.7
	Nervous	13	6.8

Colleague relationship	Harmonious	76	40.0
	Commonly	52	27.4
	Nervous	2	1.1
	No job	60	31.6

Working conditions	Regular job	95	50.0
	Irregular work	33	17.4
	Jobless	62	32.6

**Table 2:** Analysis of the prevalence of epilepsy among the subjects.

Prevalence		Cases (*n*)	Percentage (%)
Pathogeny	Inheritance	20	10.5
	Structure	77	40.5
	Infected	5	2.6
	Unknown	88	46.3

Age of onset (years)	≤10	31	16.3
	11–20	79	41.6
	21–30	53	27.9
	≥31	27	14.2

Course of disease (years)	≤5	50	26.3
	6–10	62	32.6
	11–20	47	24.7
	≥21	31	16.3

Seizure type	Simple part	10	5.3
	Complex part	45	23.7
	Secondary comprehensiveness	103	54.2
	Full-scale attack	20	10.5
	Absence attack	11	5.8
	Myoclonus	1	.5

Seizure frequency	>Once a week	14	7.4
	Once a week to once a month	52	27.4
	Once a month to once a year	58	30.5
	<1 time/year	66	34.7

No attack months after treatment	Within 1 month	65	34.2
	January to June	44	23.2
	July to December	18	9.5
	13–24 months	27	14.2
	≥25 months	36	18.9

Intractable	Yes	43	22.6
	No	147	77.4

Number of drugs used	0 species	4	2.1
	1	86	45.3
	2 kinds	87	45.8
	3 kinds	13	6.8

**Table 3:** Analysis of anxiety, depression, and sleep of the subjects.

Items		Cases (n)	Percentage (%)
Anxious	Nothing	124	65.3
	Light	43	22.6
	Moderate	15	7.9
	Heavy	8	4.2

Depressed	Normal	135	71.1
	May be depressed	44	23.2
	Depressed	8	4.2
	Severe depression	3	1.6

Drowsiness	Normal	136	71.6
	Drowsiness	37	19.5
	Excessive drowsiness	15	7.9
	Dangerous sleepiness	2	1.1

Sleep quantity	Normal	148	77.9
	Difference	42	22.1

**Table 4:** Dimensions and scale scores of quality of life in patients with epilepsy.

Dimension	Values
Attack worry	61.3 ± 28.3
Overall quality of life	13.8 ± 4.0
Emotional health	69.4 ± 17.5
Tiredness	50.4 ± 16.1
Cognitive function	77.2 ± 25.6
Drug effects	35.4 ± 18.2
Social activities	63.2 ± 16.3
Total score of epilepsy life scale	29.6 ± 7.5

**Table 5:** Correlation between quality of life and demographic factors in patients with epilepsy.

Items		Seizure anxiety	Life satisfaction	Emotional health	Energy fatigue	Cognitive function	Drug effect	Social activity	Total score
Gender	Male	65.6 ± 29.4	65.6 ± 29.4	65.6 ± 29.4	65.6 ± 29.4	65.6 ± 29.4	65.6 ± 29.4	65.6 ± 29.4	65.6 ± 29.4
	Female	57.5 ± 26.8	57.5 ± 26.8	57.5 ± 26.8	57.5 ± 26.8	57.5 ± 26.8	57.5 ± 26.8	57.5 ± 26.8	57.5 ± 26.8

*t*		1.98	0.049	1.98	0.049	1.98	0.049	1.98	0.049
*p*		2.00	0.047	2.00	0.047	2.00	0.047	2.00	0.047
Age (years)	≤25	56.6 ± 31.4	56.6 ± 31.4	56.6 ± 31.4	56.6 ± 31.4	56.6 ± 31.4	56.6 ± 31.4	56.6 ± 31.4	56.6 ± 31.4
	26–40	62.7 ± 27.1	62.7 ± 27.1	62.7 ± 27.1	62.7 ± 27.1	62.7 ± 27.1	62.7 ± 27.1	62.7 ± 27.1	62.7 ± 27.1
	≥41	64.2 ± 26.2	64.2 ± 26.2	64.2 ± 26.2	64.2 ± 26.2	64.2 ± 26.2	64.2 ± 26.2	64.2 ± 26.2	64.2 ± 26.2

*F*		1.11	0.331	1.11	0.331	1.11	0.331	1.11	0.331
*p*		0.43	0.649	0.43	0.649	0.43	0.649	0.43	0.649
Marriage	Unmarried	58.4 ± 30.9	61.6 ± 27.0	74.1 ± 23.3	58.4 ± 30.9	61.6 ± 27.0	74.1 ± 23.3	58.4 ± 30.9	61.6 ± 27.0
	Married	13.2 ± 4.24	13.9 ± 3.87	15.2 ± 3.10	13.2 ± 4.24	13.9 ± 3.87	15.2 ± 3.10	13.2 ± 4.24	13.9 ± 3.87
	Divorce	71.0 ± 15.5	68 ± 18.6	75.2 ± 14.2	71.0 ± 15.5	68 ± 18.6	75.2 ± 14.2	71.0 ± 15.5	68 ± 18.6

*F*		1.37	0.258	1.37	0.258	1.37	0.258	1.37	0.258
*p*		1.34	0.264	1.34	0.264	1.34	0.264	1.34	0.264
Education	Below junior high school	60 ± 28.7	63.7 ± 26.6	58.4 ± 31.2	60.2 ± 28.8	66.7 ± 26.7	60 ± 28.7	63.7 ± 26.6	58.4 ± 31.2
	Junior middle school	14.6 ± 2.9	14.1 ± 3.6	12.4 ± 5.0	14.0 ± 4.1	12.4 ± 2.5	14.6 ± 2.9	14.1 ± 3.6	12.4 ± 5.0
	High school	64.1 ± 20.4	67.1 ± 17.7	74.2 ± 15.8	70.9 ± 15.7	73.5 ± 17.8	64.1 ± 20.4	67.1 ± 17.7	74.2 ± 15.8
	Junior College	48.6 ± 20.0	49.0 ± 15.4	51.5 ± 15.8	51.5 ± 15.2	55 ± 13.9	48.6 ± 20.0	49.0 ± 15.4	51.5 ± 15.8
	University or above	66.6 ± 30.1	77.5 ± 24.5	81.8 ± 26.6	79.6 ± 22.9	73.4 ± 22.2	66.6 ± 30.1	77.5 ± 24.5	81.8 ± 26.6

*F*		0.32	0.864	0.32	0.864	0.32	0.864	0.32	0.864
*p*		1.95	0.104	1.95	0.104	1.95	0.104	1.95	0.104
Occupation	Student	46.5 ± 30.3	65.7 ± 22.0	66.5 ± 27.4	66.3 ± 24.1	52.5 ± 29.4	46.5 ± 30.3	65.7 ± 22.0	66.5 ± 27.4
	Part-time job	15.5 ± 3.97	13.3 ± 3.65	13.3 ± 4.01	15.5 ± 3.71	13.9 ± 3.97	15.5 ± 3.97	13.3 ± 3.65	13.3 ± 4.01
	Full-time job	68.7 ± 11.5	66.9 ± 21.3	70.5 ± 17.7	64.6 ± 17.0	68.9 ± 17.2	68.7 ± 11.5	66.9 ± 21.3	70.5 ± 17.7
	Retired	44.2 ± 15.9	53.4 ± 14.7	51.2 ± 16.6	44.6 ± 17.4	50.2 ± 15.3	44.2 ± 15.9	53.4 ± 14.7	51.2 ± 16.6
	Unemployed	79.0 ± 18.3	73.8 ± 26.0	80.1 ± 25.7	71.0 ± 35.2	72.3 ± 26.2	79.0 ± 18.3	73.8 ± 26.0	80.1 ± 25.7

*F*		3.45	0.010	3.45	0.010	3.45	0.010	3.45	0.010
*p*		1.53	0.195	1.53	0.195	1.53	0.195	1.53	0.195
Smoke	Yes	60.6 ± 28.2	69.6 ± 28.4	60.6 ± 28.2	69.6 ± 28.4	60.6 ± 28.2	69.6 ± 28.4	60.6 ± 28.2	69.6 ± 28.4
	No	13.6 ± 4.05	14.9 ± 2.54	13.6 ± 4.05	14.9 ± 2.54	13.6 ± 4.05	14.9 ± 2.54	13.6 ± 4.05	14.9 ± 2.54

*t*		1.10	0.272	1.10	0.272	1.10	0.272	1.10	0.272
*p*		1.13	0.258	1.13	0.258	1.13	0.258	1.13	0.258
Drink	Yes	59.4 ± 27.9	82.8 ± 24.2	59.4 ± 27.9	82.8 ± 24.2	59.4 ± 27.9	82.8 ± 24.2	59.4 ± 27.9	82.8 ± 24.2
	No	13.8 ± 3.93	12.7 ± 4.42	13.8 ± 3.93	12.7 ± 4.42	13.8 ± 3.93	12.7 ± 4.42	13.8 ± 3.93	12.7 ± 4.42

*t*		3.15	0.002	3.15	0.002	3.15	0.002	3.15	0.002
*p*		1.02	0.309	1.02	0.309	1.02	0.309	1.02	0.309
Economic situation	Good	55.7 ± 30.1	63.8 ± 26.5	58.3 ± 30.8	55.7 ± 30.1	63.8 ± 26.5	58.3 ± 30.8	55.7 ± 30.1	63.8 ± 26.5
	Moderate	13.4 ± 3.94	13.8 ± 3.95	13.7 ± 4.09	13.4 ± 3.94	13.8 ± 3.95	13.7 ± 4.09	13.4 ± 3.94	13.8 ± 3.95
	Bad	76.7 ± 14.3	69.8 ± 15.8	65.5 ± 20.7	76.7 ± 14.3	69.8 ± 15.8	65.5 ± 20.7	76.7 ± 14.3	69.8 ± 15.8

*F*		1.22	0.296	1.22	0.296	1.22	0.296	1.22	0.296
*p*		0.10	0.902	0.10	0.902	0.10	0.902	0.10	0.902
Family situation	Harmonious	65.1 ± 28.2	57.2 ± 26.7	47.1 ± 31.9	65.1 ± 28.2	57.2 ± 26.7	47.1 ± 31.9	65.1 ± 28.2	57.2 ± 26.7
	Commonly	13.6 ± 4.15	13.9 ± 3.70	14.5 ± 3.79	13.6 ± 4.15	13.9 ± 3.70	14.5 ± 3.79	13.6 ± 4.15	13.9 ± 3.70
	Nervous	72.7 ± 16.5	65.5 ± 17.6	60 ± 18.6	72.7 ± 16.5	65.5 ± 17.6	60 ± 18.6	72.7 ± 16.5	65.5 ± 17.6

*F*		3.41	0.035	3.41	0.035	3.41	0.035	3.41	0.035
*p*		0.38	0.686	0.38	0.686	0.38	0.686	0.38	0.686
Colleague relationship	Harmonious	68.3 ± 27.4	61.7 ± 25.9	52.4 ± 28.4	68.3 ± 27.4	61.7 ± 25.9	52.4 ± 28.4	68.3 ± 27.4	61.7 ± 25.9
	Commonly	13.4 ± 4.25	13.1 ± 3.50	14.6 ± 3.95	13.4 ± 4.25	13.1 ± 3.50	14.6 ± 3.95	13.4 ± 4.25	13.1 ± 3.50
	Nervous	71.9 ± 16.8	67.2 ± 19.0	68.5 ± 15.2	71.9± 16.8	67.2 ± 19.0	68.5 ± 15.2	71.9 ± 16.8	67.2 ± 19.0

*F*		5.68	0.004	5.68	0.004	5.68	0.004	5.68	0.004
*p*		2.09	0.126	2.09	0.126	2.09	0.126	2.09	0.126
Job situation	Regular job	68.0 ± 25.6	59.0 ± 30.0	51.9 ± 28.8	68.0 ± 25.6	59.0 ± 30.0	51.9 ± 28.8	68.0 ± 25.6	59.0 ± 30.0
	No fixed work	13.5 ± 4.07	13.1 ± 3.70	14.5 ± 3.90	13.5 ± 4.07	13.1 ± 3.70	14.5 ± 3.90	13.5 ± 4.07	13.1 ± 3.70
	Jobless	70.6 ± 17.7	68.1 ± 20.7	68.1 ± 15.2	70.6 ± 17.7	68.1 ± 20.7	68.1 ± 15.2	70.6 ± 17.7	68.1 ± 20.7

*F*		6.55	0.002	6.55	0.002	6.55	0.002	6.55	0.002
*p*		1.84	0.162	1.84	0.162	1.84	0.162	1.84	0.162

**Table 6:** Correlation between quality of life and epilepsy conditions in patients with epilepsy.

Items		Seizure anxiety	Life satisfaction	Emotional health	Energy fatigue	Cognitive function	Drug effect	Social activity	Total score
Pathogeny	Inheritance	61.3 ± 28.0	57.4 ± 27.6	64.9 ± 40.4	64.3 ± 28.3	61.3 ± 28.0	57.4 ± 27.6	64.9 ± 40.4	64.3 ± 28.3
	Structure	13.5 ± 4.52	14.0 ± 3.25	13.4 ± 2.94	13.6 ± 4.47	13.5 ± 4.52	14.0 ± 3.25	13.4 ± 2.94	13.6 ± 4.47
	Infected	71.4 ± 14.4	67.8 ± 19.8	74.4 ± 9.20	70.0 ± 16.3	71.4 ± 14.4	67.8 ± 19.8	74.4 ± 9.20	70.0 ± 16.3
	Unknown	46.0 ± 16.0	50.2 ± 16.7	48.8 ± 10.7	51.6 ± 15.9	46.0 ± 16.0	50.2 ± 16.7	48.8 ± 10.7	51.6 ± 15.9

*F*		0.84	0.474	0.84	0.474	0.84	0.474	0.84	0.474
*p*		0.19	0.901	0.19	0.901	0.19	0.901	0.19	0.901
Age of onset	≤10	52.6 ± 31.6	59.2 ± 29.7	65.1 ± 24.6	69.4 ± 24.6	52.6 ± 31.6	59.2 ± 29.7	65.1 ± 24.6	69.4 ± 24.6
	11–20	13.6 ± 4.23	13.0 ± 3.89	14.5 ± 3.42	14.5 ± 4.66	13.6 ± 4.23	13.0 ± 3.89	14.5 ± 3.42	14.5 ± 4.66
	21–30	67.3 ± 17.3	69.8 ± 17.7	69.3 ± 17.9	70.5 ± 16.7	67.3 ± 17.3	69.8 ± 17.7	69.3 ± 17.9	70.5 ± 16.7
	≥31	47.1 ± 16.9	51.3 ± 16.4	51.1 ± 14.6	49.9 ± 17.2	47.1 ± 16.9	51.3 ± 16.4	51.1 ± 14.6	49.9 ± 17.2

*F*		2.22	0.087	2.22	0.087	2.22	0.087	2.22	0.087
*p*		1.88	0.134	1.88	0.134	1.88	0.134	1.88	0.134
Course of disease	≤5	58.4 ± 29.1	64.6 ± 26.5	60.6 ± 30.5	59.8 ± 27.6	58.4 ± 29.1	64.6 ± 26.5	60.6 ± 30.5	59.8 ± 27.6
	6–10	14.1 ± 3.83	13.3 ± 3.84	14.0 ± 4.48	13.7 ± 3.74	14.1 ± 3.83	13.3 ± 3.84	14.0 ± 4.48	13.7 ± 3.74
	11–20	68.1 ± 16.7	72.8 ± 14.0	70.9 ± 19.5	62.1 ± 19.8	68.1 ± 16.7	72.8 ± 14.0	70.9 ± 19.5	62.1 ± 19.8
	≥21	50.6 ± 15.7	53.0 ± 15.1	50.3 ± 15.6	45.0 ± 18.6	50.6 ± 15.7	53.0 ± 15.1	50.3 ± 15.6	45.0 ± 18.6

*F*		0.49	0.687	0.49	0.687	0.49	0.687	0.49	0.687
*p*		0.44	0.722	0.44	0.722	0.44	0.722	0.44	0.722
Seizure types	Simple part	47.4 ± 25.5	55.3 ± 30.0	62.8 ± 28.4	68.7 ± 26.3	68.1 ± 21.9	47.4 ± 25.5	55.3 ± 30.0	62.8 ± 28.4
	Complex part	14.7 ± 5.17	12.9 ± 3.64	13.9 ± 4.06	14.0 ± 4.30	14.2 ± 2.67	14.7 ± 5.17	12.9 ± 3.64	13.9 ± 4.06
	Secondary comprehensiveness	67.2 ± 16.5	68.7 ± 17.3	69.1 ± 18.3	72 ± 13.8	73.0 ± 18.7	67.2 ± 16.5	68.7 ± 17.3	69.1 ± 18.3
	Full-scale attack	48 ± 15.2	49.7 ± 16.5	51.1 ± 16.3	47.6 ± 16.0	54.9 ± 15.3	48 ± 15.2	49.7 ± 16.5	51.1 ± 16.3
	Absence attack	71.1 ± 22.1	73.0 ± 24.1	78.2 ± 26.5	83.8 ± 19.4	76.9 ± 35.9	71.1 ± 22.1	73.0 ± 24.1	78.2 ± 26.5

*F*		1.70	0.152	1.70	0.152	1.70	0.152	1.70	0.152
*p*		0.69	0.598	0.69	0.598	0.69	0.598	0.69	0.598
Seizure frequency	>1 time/week	54.0 ± 32.7	47.1 ± 29.6	64.5 ± 26.3	71 ± 23.3	54.0 ± 32.7	47.1 ± 29.6	64.5 ± 26.3	71 ± 23.3
	1 time/week to 1 time/month	12.6 ± 4.20	14.1 ± 3.40	13.8 ± 3.67	13.6 ± 4.59	12.6 ± 4.20	14.1 ± 3.40	13.8 ± 3.67	13.6 ± 4.59
	1 time/month to 1 time/year	63.7 ± 18.3	66.7 ± 19.2	69.4 ± 16.3	72.6 ± 16.5	63.7 ± 18.3	66.7 ± 19.2	69.4 ± 16.3	72.6 ± 16.5
	<1 time/year	47.7 ± 18.9	47.4 ± 16.6	52.9 ± 13.7	51.1 ± 16.9	47.7 ± 18.9	47.4 ± 16.6	52.9 ± 13.7	51.1 ± 16.9

*F*		8.33	0.000	8.33	0.000	8.33	0.000	8.33	0.000
*p*		0.58	0.631	0.58	0.631	0.58	0.631	0.58	0.631
No attack months after treatment	≤1 month	48.5 ± 30.2	61.7 ± 26.8	70.8 ± 25.5	67.1 ± 23.7	74.4 ± 22.0	48.5 ± 30.2	61.7 ± 26.8	70.8 ± 25.5*∗*
	1–6 months	13.9 ± 3.60	13.5 ± 3.53	13.6 ± 4.86	13.5 ± 4.19	14.0 ± 4.63	13.9 ± 3.60	13.5 ± 3.53	13.6 ± 4.86
	7–12 months	66.2 ± 18.9	70.2 ± 16.5	68.4 ± 15.2	68.4 ± 15.3	75.3 ± 17.6	66.2 ± 18.9	70.2 ± 16.5	68.4 ± 15.2
	13–24 months	47.4 ± 16.8	52.1 ± 13.8	51.5 ± 13.7	50.3 ± 18.5	53.1 ± 16.4	47.4 ± 16.8	52.1 ± 13.8	51.5 ± 13.7
	≥25 months	71.4 ± 25.4	77.9 ± 26.3	83.1 ± 25.6	74.2 ± 24.9	85.6 ± 23.7	71.4 ± 25.4	77.9 ± 26.3	83.1 ± 25.6

*F*		6.75	0.000	6.75	0.000	6.75	0.000	6.75	0.000
*p*		0.10	0.981	0.10	0.981	0.10	0.981	0.10	0.981
Intractable	Yes	45.0 ± 28.9	66.0 ± 26.3	45.0 ± 28.9	66.0 ± 26.3	45.0 ± 28.9	66.0 ± 26.3	45.0 ± 28.9	66.0 ± 26.3
	No	13.9 ± 3.58	13.7 ± 4.09	13.9 ± 3.58	13.7 ± 4.09	13.9 ± 3.58	13.7 ± 4.09	13.9 ± 3.58	13.7 ± 4.09

*t*	4.48	0.000	4.48	0.000	4.48	0.000	4.48	0.000	
*p*	0.34	0.731	0.34	0.731	0.34	0.731	0.34	0.731	
Number of drugs used	One	64.8 ± 28.2	60.6 ± 27.3	42.8 ± 31.9	64.8 ± 28.2	60.6 ± 27.3	42.8 ± 31.9	64.8 ± 28.2	60.6 ± 27.3*∗*
	Two	14.0 ± 3.55	13.2 ± 4.34	14.6 ± 3.08	14.0 ± 3.55	13.2 ± 4.34	14.6 ± 3.08	14.0 ± 3.55	13.2 ± 4.34
	Three	70.8 ± 16.8	67.9 ± 19.0	69.2 ± 12.7	70.8 ± 16.8	67.9 ± 19.0	69.2 ± 12.7	70.8 ± 16.8	67.9 ± 19.0

*F*		3.52	0.032	3.52	0.032	3.52	0.032	3.52	0.032
*p*		1.26	0.286	1.26	0.286	1.26	0.286	1.26	0.286

**Table 7:** Correlation analysis of anxiety, depression, sleep, and quality of life.

Items	Anxiety	Somnolence	Depression	Sleep quality	Health status
Somnolence	0.333^*∗∗*^				
Depression	0.675^*∗∗*^	0.201^*∗∗*^			
Sleep quality	0.520^*∗∗*^	0.343^*∗∗*^	0.569^*∗∗*^		
Health status	−0.407^*∗∗*^	−0.225^*∗∗*^	−0.342^*∗∗*^	−0.264^*∗∗*^	
Quantity of life	−0.645^*∗∗*^	−0.266^*∗∗*^	−0.599^*∗∗*^	−0.407^*∗∗*^	0.513^*∗∗*^

^*∗∗*^ *P* < 0.01

**Table 8:** Path coefficient test of structural equation model for influencing factors of quality of life in patients with epilepsy.

Pathway			Nonstandard path coefficient	S.E.	C.R.	*P*	Standard path coefficient
Somnolence	<---	Sleep quality	0.431	0.086	5.012	*∗∗∗*	0.343
Depression	<---	Sleep quality	0.982	0.103	9.522	*∗∗∗*	0.569
Anxious	<---	Depressed	0.432	0.048	9.007	*∗∗∗*	0.559
Anxious	<---	Sleep quality	0.191	0.086	2.213	0.027	0.143
Anxious	<---	Drowsiness	0.183	0.058	3.175	0.001	0.172
Health status	<---	Anxious	−2.031	0.332	−6.122	*∗∗∗*	−0.407
Quality of life	<---	Depressed	−0.343	0.088	−3.924	*∗∗∗*	−0.266
Quality of life	<---	Anxious	−0.591	0.119	−4.958	*∗∗∗*	−0.355
Quality of life	<---	Health	0.093	0.018	5.101	*∗∗∗*	0.280

*∗∗∗P* < 0.001

**Table 9:** Fitting results of structural equation model.

Indicators	Reference standard	This model
*χ * ^2^/df	<3	0.776
P	＞0.05	0.589
GFI	＞0.90	0.992
CFI	＞0.90	0.999
RMSEA	＜0.080	0.001
IFI	＞0.90	0.999
AGFI	＞0.90	0.972
PGFI	＞0.50	0.483
PNFI	＞0.50	0.490

## Data Availability

The data sets used and/or analyzed during the current study are available from the corresponding author on reasonable request.

## Abbreviations

QOLIE-31: Quality of life in epilepsy Inventory-31
PSQI: Pittsburgh sleep quality index
ESS: Epworth sleepiness scale
HAMD-17: Hamilton depression scale-17
GAD-7: Generalized anxiety disorder-7.

## References

[1] Davis P. J., Holmes D., Waltho J. P., Staniforth R. A. (2015). Limited proteolysis reveals that amyloids from the 3D domain-swapping cystatin B have a non-native *β*-sheet topology. *Journal of Molecular Biology*.

[2] Ngugi A. K., Bottomley C., Fegan G. (2014). Premature mortality in active convulsive epilepsy in rural Kenya: causes and associated factors. *Neurology*.

[3] China antiepileptic Association (2015). China. antiepileptic Association. *guidelines for clinical diagnosis and treatment: epilepsy volume*.

[4] Fiest K. M., Dykeman J., Patten S. B. (2013). Depression in epilepsy: a systematic review and meta-analysis. *Neurology*.

[5] Epilepsy (1862). Its symptoms, treatment, and relation to other chronic convulsive diseases. *Br Foreign Med Chir Rev*.

[6] (1995). The world health organization quality of life assessment (WHOQOL): position paper from the world health organization. *Social Science & Medicine*.

[7] Cramer J. A., Perrine K., Devinsky O., Bryant-Comstock L., Meador K., Hermann B. (1998). Development and cross-cultural translations of a 31-item quality of life in epilepsy inventory. *Epilepsia*.

[8] Montgomery S. A. (2006). Major depressive disorders: clinical efficacy and tolerability of agomelatine, a new melatonergic agonist. *European Neuropsychopharmacology*.

[9] He X., Li C., Qian J. (2010). Reliability and validity of generalized anxiety scale in general hospitals. *Shanghai archives of psychiatry*.

[10] Liu X. (1996). Tang maoqin: reliability and validity of Pittsburgh sleep quality index. *Chinese Journal of psychiatry*.

[11] Johns M. W. (1993). Daytime sleepiness, snoring, and obstructive sleep apnea. *Chest*.

[12] Räty L., Hamrin E., Söderfeldt B. (2009). Quality of life in newly-debuted epilepsy. An empirical study. *Acta Neurologica Scandinavica*.

[13] Nubukpo P., Clément J. P., Houinato D. (2004). Psychosocial issues in people with epilepsy in Togo and Benin (West Africa) II: quality of life measured using the QOLIE-31 scale. *Epilepsy and Behavior*.

[14] Djibuti M., Shakarishvili R. (2003). Influence of clinical, demographic, and socioeconomic variables on quality of life in patients with epilepsy: findings from Georgian study. *Journal of Neurology, Neurosurgery & Psychiatry*.

[15] Zhang Y., sun M. (2015). Investigation and analysis of quality of life and its influencing factors in adult patients with epilepsy. *Journal of cardio cerebrovascular diseases of integrated traditional Chinese and Western medicine*.

[16] Wang F. L., Gu X. M., Hao B. Y., Wang S., Chen Z. J., Ding C. Y. (2017). Influence of marital status on the quality of life of Chinese adult patients with epilepsy. *Chinese Medical Journal*.

[17] Thabit M. N., Sayed M. A., Ali M. M. (2018). Evaluation of knowledge about epilepsy and attitudes towards patients with epilepsy among university students in Upper Egypt. *Epilepsy Research*.

[18] Zhao Y., Wu H., Li J. (2011). Quality of life and related factors in adult patients with epilepsy in China. *Epilepsy and Behavior*.

[19] Holland P., Lane S., Whitehead M., Marson A. G., Jacoby A. (2009). Labor market participation following onset of seizures and early epilepsy: findings from a UK cohort. *Epilepsia*.

[20] Dworetzky B. A., Bromfield E. B., Townsend M. K., Kang J. H. (2010). A prospective study of smoking, caffeine, and alcohol as risk factors for seizures or epilepsy in young adult women: data from the Nurses’ Health Study II. *Epilepsia*.

[21] Cheng J., Wang tongge, Cheng J. (2007). Investigation and analysis of influencing factors of quality of life in adult patients with epilepsy. *Chinese Journal of continuing medical education*.

[22] Wang Li (2017). Effect of follow-up nursing on anxiety, depression and quality of life of adult patients with epilepsy. *China continuing medical education*.

[23] Stafstrom C. E., Lynch M., Sutula T. P. (2000). Consequences of epilepsy in the developing brain: implications for surgical management. *Seminars in Pediatric Neurology*.

[24] Li K., He J., Jiang X. (2015). Investigation on quality of life and its influencing factors of epilepsy patients in rural areas of Gansu Province. *Chinese Journal of neuropsychiatric diseases*.

[25] Fawale M. B., Owolabi M. O., Ogunniyi A. (2014). Effects of seizure severity and seizure freedom on the health-related quality of life of an African population of people with epilepsy. *Epilepsy and Behavior*.

[26] Chen E., Sajatovic M., Liu H. (2018). Demographic and clinical correlates of seizure frequency: findings from the managing epilepsy well network database. *Journal of Clinical Neurology*.

[27] Alexander H. B., Broshek D. K., Quigg M. (2018). Quality of life in adults with epilepsy is associated with anticonvulsant polypharmacy independent of seizure status. *Epilepsy and Behavior*.

[28] Nagarathnam M., Vengamma B., Shalini B., Latheef S. (2017). Stigma and polytherapy: predictors of quality of life in patients with epilepsy from south India. *Annals of Indian Academy of Neurology*.

[29] Wang Y., Wang H., Zhao Y. (2018). Relationship between emotional health and quality of life and suicide in epileptic patients. *China Journal of Health Psychology*.

[30] Johnson E. K., Jones J. E., Seidenberg M., Hermann B. P. (2004). The relative impact of anxiety, depression, and clinical seizure features on health-related quality of life in epilepsy. *Epilepsia*.

[31] Kwan P., Yu E., Leung H., Leon T., Mychaskiw M. A. (2009). Association of subjective anxiety, depression, and sleep disturbance with quality-of-life ratings in adults with epilepsy. *Epilepsia*.

[32] García-Morales I., Gil-Nagel A., de Rosendo J., Torres-Falcón A. (2014). [Sleep disorders and quality of life in refractory partial epilepsy: results of the SLEEP study]. *Revue Neurologique*.

[33] Buratti L., Natanti A., Viticchi G. (2018). Impact of sleep disorders on the risk of seizure recurrence in juvenile myoclonic epilepsy. *Epilepsy and Behavior*.

